# Super‐stiff guidewire or loach guidewire during percutaneous nephrolithotomy?

**DOI:** 10.1002/bco2.219

**Published:** 2023-04-28

**Authors:** Xiaobo Ding, Yuchuan Hou, Chunxi Wang, Yanbo Wang

**Affiliations:** ^1^ Department of Radiology First Hospital of Jilin University Changchun China; ^2^ Department of Urology First Hospital of Jilin University Changchun China

**Keywords:** endourology, kidney stones, loach guidewire, percutaneous nephrolithotomy (PCNL), super‐stiff guidewire

## Abstract

**Objectives:**

The objectives of this work are to compare the outcomes between loach guidewire and super‐stiff guidewire during percutaneous nephrolithotomy (PCNL) and find potential indications of different guidewires.

**Patients and methods:**

We retrospectively reviewed our institutional PCNL database from 2017 to 2021. Patients who underwent PCNL guided by loach guidewire were assigned to group A (489 patients); patients who received super‐stiff guidewire were assigned to group B (269 patients). Preoperative demographic data, intraoperative parameters, and postoperative complications were compared. The conditions and reasons of failed placement of guidewire needed readjustment were evaluated as well.

**Results:**

Preoperative demographic data and most intraoperative parameters were not statistically different between the groups. Postoperative Clavien–Dindo complications were also comparable, with low rate of complications. However, failed placement of guidewire more occurred in group A (8.2% vs. 4.0%, respectively, *p* = 0.03). Compared with the super‐stiff guidewire, the loach guidewire was easier pass/slip into any place either it be perinephric or blood vessels. In most failed group A cases and all failed group B cases, the guidewire was placed in the perirenal fat. Six patients (15%) in group A, the guidewires entered into vessels.

**Conclusions:**

Our results support that the faulty placement of loach guidewire is significantly more common compared with super‐stiff guidewire. Double confirmation is needed to prevent a major complication out of wrong dilatation whenever there is doubt about the wrong location of the guidewire.

## 1 INTRODUCTION

Percutaneous nephrolithotomy (PCNL) was first described in 1976.^1^ Such a technique has rapidly progressed from then on, becoming the first line for the treatment of large and complex kidney stones.^2^, ^3^ In the past 40 years, various innovations have emerged about PCNL including patients' position,^4^, ^5^, ^6^ anaesthesia approaches,^7^, ^8^ puncture methods,^9^, ^10^, ^11^ dilatation tools,^12^ number of tracts,^13^, ^14^, ^15^ lithotripsy instruments,^16^ and placement of tubes.^17^, ^18^, ^19^ However, the guidewire, one minor but very cruical tool used during PCNL, is still only including two types, loach guidewire and super‐stiff guidewire. Nevertheless, the different available guidewires have never been investigated looking for the better one, and their role still remains not well clarified. Some authors prefer to choose the loach guidewire during PCNL^20^, ^21^ while others routinely use the super‐stiff guidewire.^22^, ^23^

Both loach guidewire and super‐stiff guidewire have been widely used, but the indications and the outcomes associated are poorly defined. Trying to give a contribution in this field, we designed the present study, aimed to compare the outcomes between loach guidewire and super‐stiff guidewire during PCNL. The secondary outcome of the study was to assess the reasons for failed placement of guidewire and find potential indications of different guidewires.

## 2 PATIENTS AND METHODS

After institutional review board approval of the First Hospital of Jilin University, we retrospectively reviewed the records of patients who underwent PCNL from 1 May 2017 to 30 October 2021. All methods were carried out in accordance with relevant guidelines and regulations. Informed consent was obtained from the patients. The patients were excluded if they received preoperative nephrostomy. Ultrasonography, intravenous urography, and/or computed tomography scan were preoperatively used to evaluate the complexity of stones and the presence of hydronephrosis. Based on whether it was loach or super‐stiff guidewire, patients were divided into group A (loach guidewire group) and group B (super‐stiff guidewire group). Patients' demographics and characteristics, intraoperative and postoperative parameters, were recorded. The style of stones and presence of hydronephrosis were analysed, and the reasons of failed placement of guidewire were also explored. All cases were performed by one surgeon who had successfully finished more than 3000 cases of PCNL. Clavien–Dindo classification was used to grade the PCNL‐related complications.^24^

All procedures performed in studies involving human participants were obtained from the institutional ethics committee of the First Hospital of Jilin University, Changchun, China.

The procedures were performed under general anaesthesia in both groups. After a 5‐French external ureteral catheter was inserted in a retrograde fashion into the renal pelvis or the upper ureter in a lithotomy position (in order to be prepared for potential retrograde saline injection), the patient was placed in prone position on the UROSKOP Access surgery bed (Siemens, Germany). Such an X‐ray bed could help to reconfirm the needle puncture site and placement of guidewire if necessary. It can also benefit to evaluate the position of double‐J stent and postoperative immediate residual stones.

**FIGURE 1:**
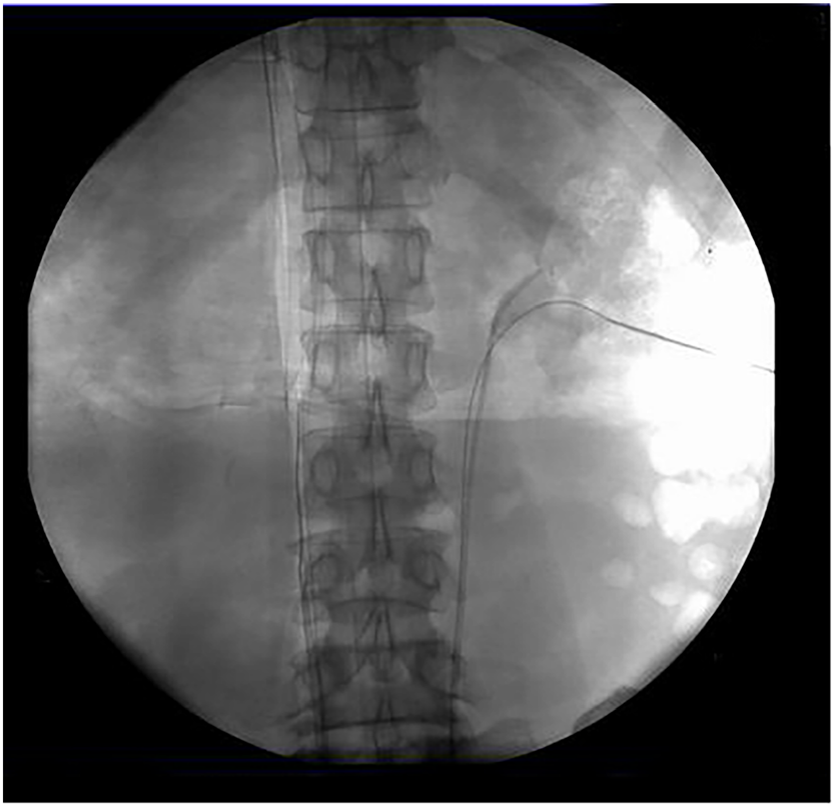
Loach guidewire was successfully placed antegrade into the ureter.

Under the guidance of ultrasound (Aloka 7, Japan), the best puncture spot was achieved using an 18‐gauge needle (Cook Inc., USA) based on the stone configuration and surgeon's preference. Then, a loach straight tip guidewire (0.032 in × 145 cm, Cook Inc., USA) or a super‐stiff J tipped guidewire (0.035 in × 145 cm, 3.5 cm flexible tip, Boston Scientific, USA) was inserted into the pelvis or coiled in the target calyx (Figure 1). The loach guidewire is the hydrophilic guidewire. Percutaneous access was established using balloon dilators (BCR Inc., USA) up to 24‐French, guided by the wire. Ultrasonic or pneumatic lithotripsy was used to fragment the stone through a rigid 20‐French nephroscope (Storz, Germany). Multiaccess was established in the same way when needed. A 5‐French double‐J stent and a 20‐French nephrostomy were routinely installed and were postoperatively removed 2 to 4 weeks and 2 to 3 days postoperatively, respectively.

Residual fragments ≤4 mm were accepted as stone free as previously described, and postoperative instant stone free rate (SFR) was evaluated at the end of the surgery based on the ultrasound and X‐ray images.^25^ The definition of a failed placement or misplacement of guidewire is that the guidewire does not enter into the collecting system, which is confirmed by endoscopy and/or fluoroscopy. Operation time was defined from the start of retrograde insertion to the completion of nephrostomy tube placement.

Results were reported by descriptive statistics. Continuous variables were reported as medians with interquartile range, and categorical variables were reported as frequencies and proportions. Continuous variables were compared using Mann–Whitney *U* test, and categorical variables were compared using chi‐square test. Subanalysis was performed to compare the failed placement of guidewires between loach and super‐stiff guidewires using chi‐square test. All *p* values were two‐tailed, and *p* < 0.05 was considered significant. Statistical analysis was performed using SPSS software package (versions 17.0, SPSS, Inc., Chicago, IL, USA).

## 3 RESULTS

The characteristics of loach guidewire group (group A) and super‐stiff guidewire group (group B) patients are reported in Table 1. There were no statistically significant differences between male/female ratio, mean age, body mass index, left/right side, ASA physical status, Guy's stone score, stone diameter, stone position, stone density, percentage of hydronephrosis, and previous surgical history.

**TABLE 1:** Patient characteristics

Parameters	Group A (loach guidewire, *n* = 489)	Group B (super‐stiff guidewire, *n* = 269)	*p* value
M/F	263/226	145/124	0.975
Age (year), median (IQR)	41.2(25–63)	41.8(24–64)	0.573
Mean BMI, kg/m^2^	24.9(20–31)	25.0(20–30)	0.647
Left/right side	258/231	141/128	0.928
ASA physical status, *n*(%)			0.489
Class 1	355(72.6)	193(71.7)	
Class 2	108(22.1)	56(20.8)	
Class 3	26(5.3)	20(7.5)	
Guy's stone score, *n*(%)			0.628
I	67(13.7)	36(13.4)	
II	125(25.6)	74(27.5)	
III	163(33.3)	78(29.0)	
IV	134(27.4)	81(30.1)	
MSD (cm), median (IQR)	3.6(2.1–9.5)	3.8(2.1–8.9)	0.723
Stone position, *n*(%)			0.260
Upper pole	88(18.0)	59(21.9)	
Middle pole	27(5.5)	19(7.1)	
Lower pole	14(2.9)	9(3.3)	
Pelvis	38(7.8)	12(4.5)	
Multiple sites	322(65.8)	170(63.2)	
Stone density (HU), median (IQR)	805(621–1132)	823(533–1237)	0.438
Hydronephrosis, yes/no	293/196	159/110	0.828
History of surgery, *n*(%)	35(7.2)	23(8.6)	0.490

Abbreviations: BMI, body mass index; F, female; IQR, interquartile range; M, male; MSD, maximum stone diameter.

Mean operation time was 43.2 min in group A and 39.5 min in group B (*p* = 0.253). Ultrasound time, fluoroscopy time, and aimed calyx were comparable in both groups (Table 2). There were no significant differences in postoperative instant SFR (86.1% and 89.2%, respectively, *p* = 0.218). However, in comparison to group B, more patients encountered failed placement of guidewire in group A (8.2% and 4.0%, respectively, *p* = 0.03). Grades I–II complications were comparable in the two cohorts (*p* = 0.59 and 0.322, respectively). Grades III–IV complications were low in both groups; 0.6% in group A and 1.1% in group B were grade III, and 0.6% in group A and 0.8% in group B were grade IV postoperative complications. No grade V Clavien complications occurred in both groups.

**TABLE 2:** Intraoperative and postoperative parameters

Parameters	Group A (loach guidewire, *n* = 489)	Group B (super‐stiff guidewire, *n* = 269)	*p* value
OT (min), median (IQR)	43.6(31–120)	41.2(29–112)	0.253
US guided access time (second), median (IQR)	17.7(10–41)	18.9(7–46)	0.475
Fluoroscopy time (second), median (IQR)	4.8(4–8)	4.3(4–7)	0.683
Aimedcalyx, *n*(%)			0.08
Upper	128(26.2)	82(30.5)	
Middle	199(40.7)	84(31.2)	
Lower	61(12.5)	39(14.5)	
Multiple	101(20.7)	64(23.8)	
Postoperative instant SFR, *n*(%)	421(86.1)	240(89.2)	0.218
Hospitalization time (day), median (IQR)	8.3(7–12)	8.6(7–13)	0.641
Failed placement of guide wire, *n*(%)	40(8.2)	11(4.0)	0.03
Confirmed by fluoroscopy	29(72.5)	9(81.8)	
Confirmed by endoscopy	11(27.5)	2(18.2)	
Clavien complications, *n*(%)			
0	412(84.3)	226(84.0)	0.931
I	45(9.2)	28(10.4)	0.590
Bleeding	19(3.9)	6(2.2)	0.069
Fever	26(5.3)	22(8.2)	
II	26(5.3)	10(3.7)	0.322
Blood transfusion	5(1.0)	3(1.1)	0.486
Infection requiring additional antibiotics	21(4.3)	7(2.6)	
IIIa	3(0.6)	3(1.1)	0.456
IIIb	0	0	
IVa	2(0.4)	1(0.4)	0.938
IVb	1(0.2)	1(0.4)	0.668
V	0	0	

Abbreviations: IQR, interquartile range; OT, operating time; SFR, stone free rate; US, ultrasound.

**FIGURE 2:**
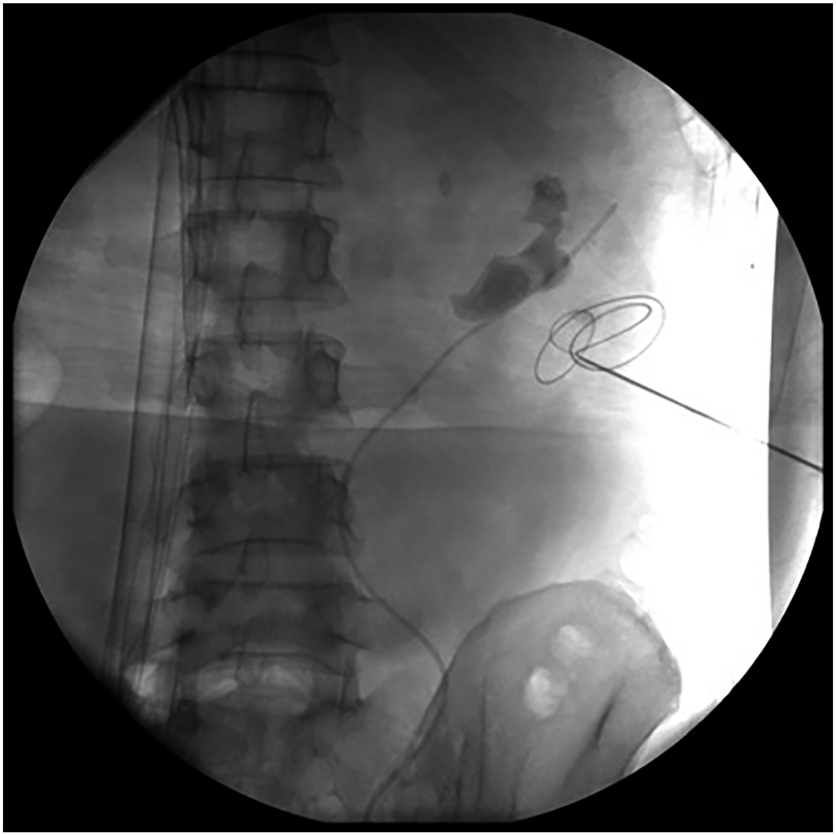
Loach guidewire was placed in perinephric space.

**FIGURE 3:**
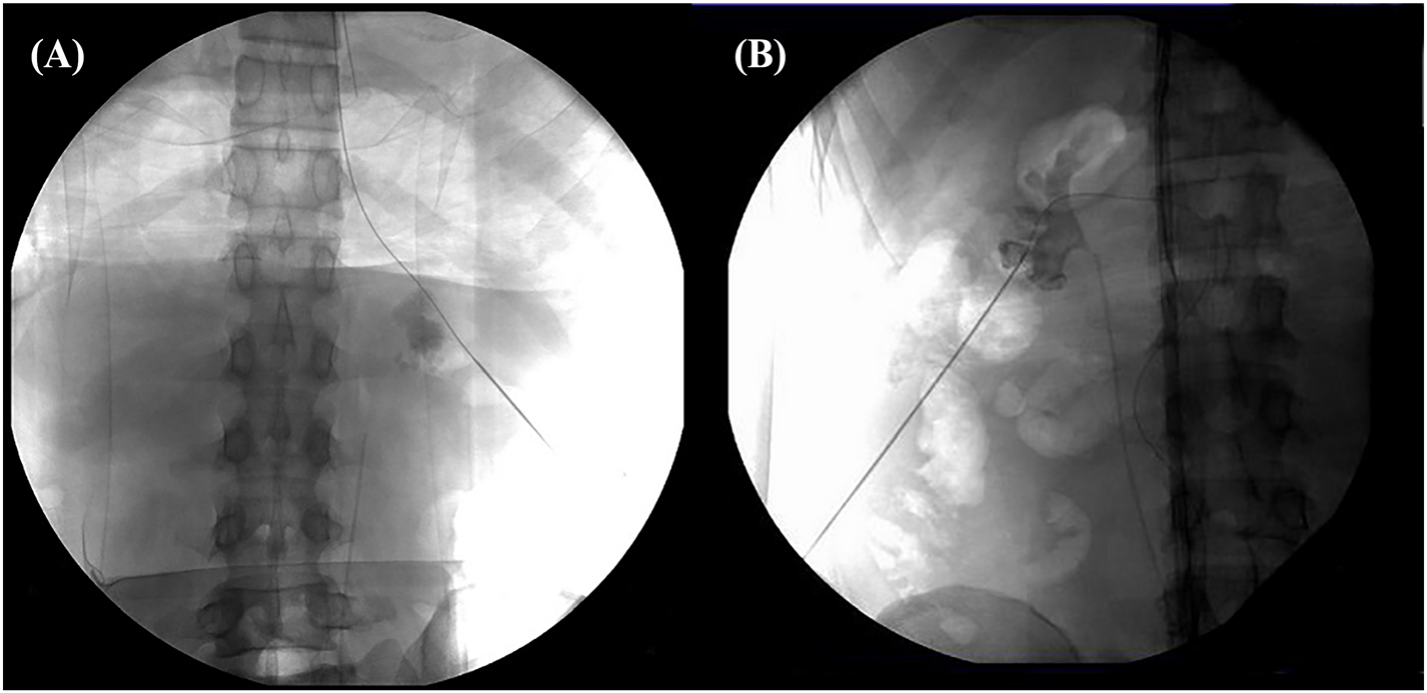
Loach guidewire was misplaced into inferior vena cava.

Forty patients in group A and 11 patients in group B encountered failed placement of guidewire (Table 3). Further analysis has shown that type of stones or presence of hydronephrosis was not significantly different between both group A and B (*p* = 0.828 and 0.609, respectively). In group B, all wires were located in the perirenal space in the failed cases (Figure 2). However, guidewires were located in the perirenal space in 85.0% failed patients, and they misentered into blood vessels in 15% patients in group A (Figure 3A,B).

**TABLE 3:** Failed placement of guidewire

Parameters	Group A (loach guidewire, *n* = 40)	Group B (super‐stiff guidewire, *n* = 11)	*p* value
Style of stones			0.828
Staghorn stones, *n*(%)	27(67.5)	8(72.7)	
Calyceal stones, *n*(%)	8(20.0)	1(9.1)	
Pelvic stones, *n*(%)	3(7.5)	1(9.1)	
Others	2(5.0)	1(9.1)	
Hydronephrosis, yes/no			0.609
Yes, *n*(%)	2(5.0)	1(9.1)	
No, *n*(%)	38(95.0)	10(90.9)	
Reasons of failure			
Located in peri‐kidney	34(85.0)	11(100)	
Entered into vessels	6(15.0)	0	

## 4 DISCUSSION

PCNL is a gold standard therapy for large and complex renal stones. However, despite both American Urological Association and European Association of Urology recommend PCNL as the first‐line therapy for patients with renal stones >20 mm, complications still occur in 1–34% of patients.^2^, ^3^, ^26^, ^27^ How to decrease the rate of complications, especially severe renal bleeding, is still challenging.

Puncture, dilatation, and fragmentation are three main and crucial steps during PCNL, among which puncture and dilatation are more important. Two kinds of guidewires are generally used after successful puncture, soft loach guidewire and super‐stiff guidewire (soft tip and stiff body).^28^, ^29^ Different surgeons prefer to choose different guidewires; however, the comparison between them is limited.

In this study, we found no differences in using the two aforementioned guidewires. Postoperative instant SFR were more than 86%, and most complications were grades I and II in the two groups.

Nineteen patients (3.9%) encountered bleeding in group A and six patients (2.2%) in group B, and 26 patients (5.3%) occurred transient fever in group A and 22 patients (8.2%) in group B (*p* = 0.069). Five patients (1.0%) needed blood transfusion in group A and three patients (1.1%) in group B, and 21 patients (4.3%) required additional antibiotics in group A and seven patients (2.6%) in group B (*p* = 0.486). Complications of grades III–IV were lower than 1% in the two groups, and all the patients discharged safely. No grade V complications occurred in any cohort.

Notably, failed placement of guidewire more occurred in group A than that in group B. Subanalysis demonstrated that staghorn stones and nonhydronephrosis were easier to yield to failed placement of guidewire, regardless the use of loach or super‐stiff guidewires. X‐ray confirmed that guidewires were located in perirenal space in all cases in group B and 85% in group A. In group A, 15% entered into blood vessels. The possible reason, in our opinion, was because of the limited space in renal collecting system for staghorn stones and kidney without hydronephrosis, which made the needle or guidewires out of the collecting system. Failing to firmly fix the needle or guidewires during the patient breath was another reason.

Intravenous misplacement of nephrostomy and double‐J stent were serious complications. Chen and colleagues reviewed 4148 patients with urolithiasis who underwent PCNL and found that 0.5% experienced misplacement of a nephrostomy.^30^ Based on our data, faulty placement of guidewire can occur in using both loach guidewire and super‐stiff guidewire independent of the type of stones or the existence of hydronephrosis. However, compared with the super‐stiff guidewire, it was easier for the loach guidewire to pass/slip into any place be it perinephric or blood vessels due to the existing small calibre and hydrophilic and flexible nature inherent to loach guidewire.

To our knowledge, this is the first study that directly compare the use of loach and super‐stiff guidewires during PCNL, showing that both of them are safe and effective, while faulty placement of loach guidewire is significantly more common compared with super‐stiff guidewire. However, as a retrospective study, the selection bias was inherent and further prospective studies are needed. Although the surgeon in this study is experienced, the learning curve is still a confounding factor. It is better to list the type of stones. What is more, change of ipsilateral renal function and final SFR should also be evaluated. The slight size difference between the guidewires (0.032 vs. 0.035 in) could also be a confounding issue with a thinner wire prone to bending and dislodgement. The imbalance in the number of participants is indeed an objective fact, which is also one of the shortcomings in this study.

In conclusion, the faulty placement of loach guidewire is significantly more common compared with super‐stiff guidewire. Hence, any endourologist using loach guidewire should know its inherent flexible quality. Double confirmation is needed using contrast injection/fluoroscopy to prevent a major complication out of wrong dilatation whenever there is doubt about the wrong location of the guidewire. Prospective studies are needed to confirm these findings.

## CONFLICT OF INTEREST

None of the authors have any disclosures or conflicts of interest to report.

## AUTHOR CONTRIBUTIONS

Xiaobo Ding designed and drafted the manuscript. Yuchuan Hou and Chunxi Wang concepted and revised the manuscript. Yanbo Wang concepted, designed and revised the manuscript.
